# Cancers primitifs oto-rhino-laryngologiques et cervico-maxillo-faciaux: aspects épidémiologiques et histopathologiques

**DOI:** 10.11604/pamj.2016.25.47.9953

**Published:** 2016-09-29

**Authors:** Bathokedeou Amana, Winga Foma, Essobozou Pegbessou, Haréfétéguéna Bissa, Saliou Adam, Essobiziou Amana, Koffi Amégbor, Essohanam Boko, Eyawèlohn Kpemissi

**Affiliations:** 1 Service d’ORL et Chirurgie Cervico-maxillo-faciale du CHU Sylvanus Olympio de Lomé, Togo; 2 Laboratoire d’Anatomie Pathologique du CHU Sylvanus Olympio de Lomé, Togo; 3 Service d’ORL du CHU campus de Lomé, Togo

**Keywords:** Epidémiologie, cancer, ORL, Togo, Epidemiology, Cancer, ORL, Togo

## Abstract

**Introduction:**

Notre objectif a été d'établir le panorama des cancers primitifs oto-rhino-laryngologiques et cervico-maxillo-faciaux dans un service de référence au Togo.

**Méthodes:**

Il s’est agi d’une étude rétrospective descriptive, portant sur les cancers diagnostiqués dans le service d’ORL et de chirurgie cervico-maxillo-faciale du CHU Sylvanus Olympio de Lomé. Elle a été réalisée sur une période de 10 ans (1^er^ Janvier 2005 au 31 Décembre 2014).

**Résultats:**

Les cancers ORL et cervico-maxillo-faciaux représentaient 0,48% des consultations et 15,3% de l’ensemble des tumeurs ORL. L’âge moyen des patients était de 47 ans, avec des extrêmes de 3 mois et 86 ans. On notait une prédominance masculine; la sex-ratio était de 1,45. L’alcoolotabagisme prédominait dans le cancer du larynx. Les cancers des voies aérodigestives supérieures (VADS) ont représenté 64,8%, avec une prédominance des cancers de la cavité buccale (36,2% des VADS), suivi des cancers de l’oropharynx (18,5% des VADS) puis des cancers du larynx (18,1% des VADS). Les adénopathies cervicales malignes primitives représentaient 18%. Les lésions les plus rares étaient les cancers de l’oreille et du tissu osseux maxillo-mandibulaire (2,24% chacun). L’histologie était dominée par le carcinome épidermoïde (61,4%) suivi du lymphome non hodgkinien (23,2%).

**Conclusion:**

Les cancers ORL et cervico-maxillo-faciaux sont fréquents au Togo et diagnostiqués à tout âge. Les cancers prédominants sont ceux de la cavité buccale, du pharynx et les adénopathies cervicales malignes primitives.

## Abstract (en)

**Introduction:**

Establish the panorama of primitive oto-rhino-laryngology and cervico-maxillofacial tumors diagnosed at a reference center in Togo.

**Methods:**

We conducted a retrospective, descriptive study of cancers diagnosed at the ORL and cervico-maxillofacial surgery department of the CHU Sylvanus Olympio of Lomé. It was conducted over a period of 10 years (1 January 2005 - 31 December 2014).

**Results:**

ORL and cervico-maxillofacial tumors account for 0.48% of consultations and 15.3% of all ORL tumors. The average age of patients was 47 years, ranging from 3 months to 86 years. We noted a male predominance; the sex ratio was 1.45. Drinking alcohol and smoking tobacco prevailed in the cancer of the larynx. Upper aerodigestive tract (UAT) tumors accounted for 64,8%, with a prevalence of cancers of the oral cavity (36,2% of UAT), followed by cancers of the oropharynx (18,5% of UAT) and finally by cancers of the larynx (18,1% of UAT). Primary malignant cervical adenopathies accounted for 18%. The rarest lesions were cancers of the ear and of maxillomandibular bone tissue (2.24% each). Histology was dominated by squamous cell carcinoma (61.4%) followed by non-Hodgkin lymphoma (23.2%).

**Conclusion:**

ORL and cervico-maxillofacial tumors are frequent in Togo and can be diagnosed at any age. The predominant tumor types reported are those of the oral cavity, pharynx and primary malignant cervical adenopathies.

## Introduction

Les cancers de la sphère oto-rhino-laryngologique (ORL) et cervico-maxillo-faciale (CMF) sont des tumeurs malignes situées au carrefour des voies destinées à de grandes fonctions de l’organisme telles l’alimentation, la respiration et la communication. Les cancers ORL sont essentiellement les cancers des voies aérodigestives supérieures (VADS) développés aux dépens de la cavité buccale, du pharynx, du larynx et des cavités nasosinusiennes. Ils sont estimés à environ 500 000 nouveaux cas tous les ans à travers le monde. Dans les pays industrialisés, leur genèse est largement favorisée par un alcoolotabagisme [1]. Les cancers ORL et CMF sont dominés par les carcinomes épidermoïdes [2]. Il n’est pas aisé d’identifier des études publiées sur l’ensemble des cancers ORL et CMF en Afrique au sud du Sahara. L’objectif de ce travail est d’établir le panorama des cancers ORL et CMF dans un service de référence au Togo.

## Méthodes

Il s’est agi d’une étude rétrospective descriptive portant sur tous les cancers diagnostiqués dans le service d’ORL et de chirurgie cervico-maxillo-faciale (CCMF) du CHU Sylvanus Olympio (CHU S.O.) de Lomé du 1^er^ Janvier 2005 au 31 Décembre 2014, soit une période de 10 ans. Les données ont été collectées à partir des dossiers des patients et du registre de résultats d’examen histologique des pièces opératoires et des biopsies analysées par le Laboratoire d’Anatomie Pathologique du CHU S.O. dans la majorité des cas et quelques fois dans un laboratoire privé à Lomé et en France. Les techniques d’examen histologique utilisées au CHU S.O ont été l’examen anatomopathologique conventionnel et les techniques histochimiques. Les patients présentant une lésion, même d’allure maligne mais sans document histologique ainsi que les patients présentant des métastases ORL et CMF venant de cancers non ORL et CMF n’ont pas été inclus dans cette étude. Les données ont été saisies et analysées à l’aide du Logiciel Epi-Info 7; les comparaisons des caractéristiques étudiées ont été faites à partir des tests d’homogénéité de Chi2 ou le Risque Relatif (RR) lorsque les séries présentent chacune au plus deux modalités. Les décisions ont été prises avec un risque a de 5%.

## Résultats

**Figure 1:**
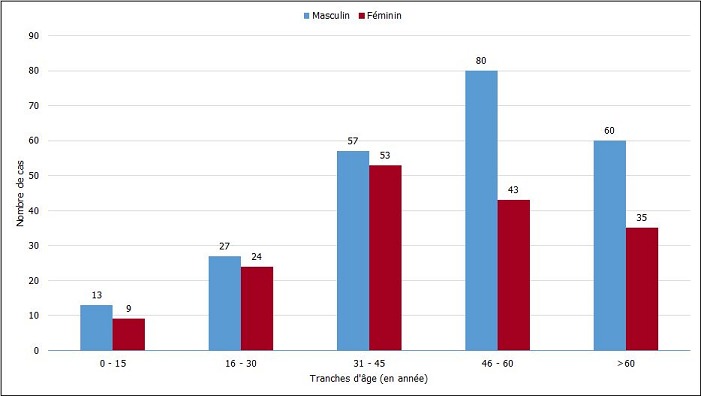
Répartition des patients atteints de cancers ORL et CMF selon le sexe et l’âge

Au cours de la période d’étude, 401 cas de cancer primitif ORL et CMF ont été histologiquement diagnostiqués. Les cancers ORL et CMF représentaient 0,48% des consultations et 15,3% de l’ensemble des tumeurs ORL et CMF. La fréquence moyenne était de 40,1 cas par an avec des extrêmes de 26 et 76 cas. Le sexe féminin était atteint dans 164 cas (41%) et le sexe masculin dans 237 cas (59%) soit une sex-ratio de 1,45. L’âge moyen des patients était de 47 ans, avec des extrêmes de 3 mois et 86 ans. La tranche d’âge de 46 à 60 ans était la plus représentée. La Figure 1 montre la répartition des patients atteints de cancers ORL et CMF selon le sexe et l’âge. Répartition des patients atteints de cancers ORL et CMF en fonction des tranches d’âge. La notion d’alcoolisme a été recherchée chez 179 patients. Il a été noté 68 cas (38%) d’intoxication alcoolique dont 13 femmes (19,12 %). Les alcools les plus retrouvés chez tous les patients étaient les boissons locales alcoolisées (alcool de vin de palme et de sorgho) et la bière. La notion de tabagisme a été recherchée chez 176 patients. Il a été noté 37 cas (21%) de tabagisme dont 2 femmes (5,4%). Le tabac était consommé sous forme de cigarette dans 36 cas et de poudre à priser dans un cas. L’intoxication simultanée d’alcool et de tabac a été retrouvée chez 29 patients. L’état d’hygiène de la cavité buccale a été recherché chez 138 patients. Dans ce groupe, nous avons noté 18 cas (13%) de mauvaise hygiène bucco-dentaire. La nature histologique des cancers était largement dominée par le carcinome épidermoïde (61,4%) suivi du lymphome non hodgkinien (LNH) (23,2%) puis du carcinome papillaire (3,7%) (Tableau 1). La prévalence des carcinomes épidermoïdes dans les cancers ORL et CMF augmentant avec l’âge a été une association statistiquement significative (Chi2 = 89,98 et p = 0) (Tableau 2) alors que celle des LNH dans les cancers ORL et CMF diminuant avec l’âge a été une association statistiquement significative (Chi2 = 121 et p = 0). Les VADS étaient représentées dans 260 cas (64,8%) et étaient répartis en 94 femmes (36,2%) et 166 hommes (63,8%). Cette localisation était dominée par les cancers de la cavité buccale (36,2%), suivi de l’oropharynx (18,5%) puis du larynx (18,1%) (Tableau 3). La localisation la plus fréquente du cancer en cas d’intoxication simultanée d’alcool et de tabac était le larynx (10 cas) suivi de l’oropharynx (7 cas). Parmi les cancers de la cavité buccale, nous avons noté une prédominance féminine (50 cas) de façon significative (p = 0,004), une association à la mauvaise hygiène bucco-dentaire (p = 7x10-4), et une prédominance du cancer de la gencive (40,4%) suivie de la langue mobile (27,7%). Les cancers des fosses nasales et des sinus étaient répartis entre les sinus maxillaires (18 cas), les fosses nasales (17 cas), les sinus ethmoïdaux (7 cas) et pyramide nasale (1 cas). Seuls 2 cas d’adénocarcinome ethmoïdal ont été retrouvés. Les cancers du cavum étaient le carcinome épidermoïde (8 cas), le lymphome (6 cas) et le carcinome indifférencié (2 cas). Dans l’oropharynx, l’amygdale palatine étaient le siège le plus fréquent avec 27 cas. Les 13 cas de cancers de l’hypopharynx étaient des carcinomes épidermoïdes; la lésion infiltrait tout l’hypopharynx chez 11 patients et siégeait à la paroi postérieure chez 2 patients. Au niveau du larynx la lésion était étendue aux trois étages chez 74,5% des patients et le carcinome épidermoïde était retrouvé dans 46 cas (98%), le LNH dans 1 cas. La prédominance masculine a été retrouvée dans les cancers des fosses nasales et sinus (72,1%), du cavum (62,5%), de l’oropharynx (70,2%), de l’hypopharynx (69,2%) et du larynx (85,1%). Les cancers de la thyroïde ont prédominé dans le sexe féminin (14 cas sur 21) répartis en carcinome papillaire (15 cas), carcinome vésiculaire (05 cas) et carcinome épidermoïde (01 cas). Les cancers des glandes salivaires étaient répartis entre la glande parotide (10 cas), la glande sous-mandibulaire (4 cas), les glandes salivaires accessoires (3 cas). Au niveau de la parotide, les types histologiques étaient l’adénocarcinome (04 cas), le carcinome épidermoïde (4 cas), le carcinome adénoïde kystique (1 cas) et le LNH (1 cas); au niveau de la glande sous-mandibulaire, nous avons retrouvé le carcinome épidermoïde (2 cas), le carcinome adénoïde kystique (1 cas) et la leucémie lymphoïde chronique (LLC) (1 cas). Les cancers de la peau cervico-faciale étaient plus retrouvés chez les femmes (7 cas) avec une prédominance du carcinome spinocellulaire (7 cas). Un patient était atteint d’albinisme oculo-cutané.

**Tableau 1:** Répartition des cancers en fonction du type histologique

	Fréquence	
Type histologique	Effectif	Pourcentage (%)
Carcinome épidermoïde	246	61,4
LNH*	93	23,2
Carcinome papillaire	15	3,7
Adénocarcinome	13	3,2
Sarcomes**	8	2
Carcinome indifférencié	5	1,2
Carcinome vésiculaire	5	1,2
Carcinome améloblastique	3	0,8
Carcinome basocellulaire	3	0,8
LLC	3	0,8
Carcinome adénoïde kystique	2	0,5
Autres ***	5	1,2
Total	401	100,0

* Regroupe les LNH à grandes cellules (69 cas), à petites cellules (8cas) et le Burkitt (16 cas)

** Regroupe le sarcome à cellules fusiformes (2 cas), le sarcome de Kaposi (3 cas), le sarcome améloblastique (1 cas) et le rhabdomyosarcome (2cas).

*** Regroupe le carcinome muco-épidermoide, le carcinome transitionnel, le neuroblastome, le Lymphome Hodgkinien et le mélanome dans 1 cas chacun.

**Tableau 2:** Prévalence des carcinomes épidermoïdes selon les tranches d’âge

Tranche d'âge (ans)	Carcinome épidermoïde		Autres Cancers ORL CMF		Total		RR/Chi2	p value; IC95%
	n	(%)	n	(%)	n	(%)		
≤ 15	0	(0)	22	(100)	22	(100)	Chi2 = 89,98	0,00
16 à 30	16	(31,4)	35	(68,6)	51	(100)		
31 à 45	58	(52,7)	52	(47,3)	110	(100)		
46 à 60	88	(71,5)	35	(28,5)	123	(100)		
>60	84	(88,4)	11	(11,6)	95	(100)		
Total	246	(61,4)	155	(38,6)	401	(100)		

La prévalence des carcinomes épidermoïdes dans les cancers ORL et CMF augmentant avec l’âge a été une association statistiquement très significative (Chi2 = 89,98 et p = 0).

**Tableau 3:** Distribution des cancers ORL et CMF selon le siège

Siège	Fréquence	
	Effectif	Pourcentage
Cavité buccale	94	23,44
Ganglions cervico-faciaux	72	18,0
Larynx	47	11,7
Oropharynx	47	11,7
Nez/Sinus	43	10,72
Thyroïde	21	5,24
Glandes salivaires	17	4,24
Cavum	15	3,74
Hypopharynx	13	3,24
Peau cervico faciale	12	2,99
Oreille	9	2,24
Mandibule	6	1,5
Maxillaire	3	0,75
Double localisation (Oropharynx + Cavum)	1	0,25
Muscles cervicaux	1	0,25
Total	401	100,0

## Discussion

La fréquence moyenne dans notre série a été de 40,1 cas par an. Ce chiffre pourrait être sous-estimé quand on sait que certains malades n’arrivent pas jusqu’à l’hôpital, car délibérément orientés vers le tradipraticien et que bon nombre de cas cliniquement suspects n’ont pu être confirmés. La prédominance masculine des cancers ORL retrouvée dans la littérature est généralement liée à la fréquence des facteurs de risque tel que l’alcoolisme et le tabagisme qui sont l’apanage du sexe masculin. De nos jours on assiste à une féminisation de ces facteurs de risque dans nos pays et l’incidence des cancers ORL diminue chez l’homme mais augmente chez la femme [3] ce qui pourrait expliquer la sex-ratio relativement basse dans notre série. L’âge moyen relativement bas dans la plupart des séries comme dans la nôtre, pourrait s’expliquer d’abord par le fait que l’intoxication alcoolotabagique est en augmentation chez les sujets jeunes et ensuite par l’espérance de vie qui est relativement peu élevée en Afrique sub-saharienne. Dolivet et al dans leur étude ont montré que l’âge de survenue des cancers des VADS est plus précoce (4^ème^, 6^ème^ décennie) pour le pic principal [4]. Bien que mal précisée, la notion d’intoxication alcoolotabagique a été associée au cancer du larynx dans notre étude. La cigarette est une cause reconnue de cancer de la cavité buccale, du pharynx et du larynx. Cette association a été retrouvée dans les séries d’Espinosa et al [5] en Espagne et de Choi et al en Corée du Sud [6]. Dans les pays industrialisés, la genèse des cancers ORL et CMF est largement dominée par un alcoolotabagisme, même si d´autres facteurs sont maintenant connus ou suspectés [1]. La mauvaise hygiène bucco-dentaire a été retrouvée dans 13% des cas avec une association prédominante aux cancers de la cavité buccale dans notre série. Dans la littérature, le rôle de la mauvaise hygiène bucco-dentaire a été reconnu dans la genèse des cancers bucco-pharyngés [1, 7]. Les cancers des VADS ont été les plus fréquents avec la cavité buccale en tête suivi du pharynx. Les cancers de l’oreille et du tissu osseux maxillo-mandibulaire sont apparus relativement plus rares. Dans la littérature, les cancers de la cavité orale et du pharynx représentent en général 75% des cancers des VADS [2]. Nous avons retrouvé une prédominance féminine dans les cancers de la cavité buccale; la plupart des auteurs ont rapporté à des degrés variables une prédominance masculine [8, 9]. De nos jours l’incidence des cancers de la cavité buccale est en forte baisse chez les hommes et en forte augmentation chez les femmes. Cette évolution est à mettre en parallèle avec la fréquence des principaux facteurs de risque [9]. Tout comme chez certains auteurs [10], les cancers des fosses nasales et des sinus sont relativement rares au Togo. La notion d’adénocarcinsome de l’ethmoïde chez des travailleurs du bois rapportée dans la littérature n’a pas été retrouvée dans notre étude, peut-être parce que nos menuisiers sont souvent installés en plein air. Le cancer du cavum au Togo est rare par rapport aux séries du bassin méditerranéen et du Sud-Est asiatique [11, 12]. L’explication pourrait en être la situation du Togo dans une zone à faible incidence de cancer indifférencié du nasopharynx au regard des facteurs alimentaires et viraux généralement incriminés. Concernant les cancers des glandes salivaires, au Togo, contrairement aux données de la littérature [13], le carcinome épidermoïde est fréquemment retrouvé, comme en témoigne l’étude de Amana et al [14] sur les cancers de la parotide. Concernant le cancer cutané facial, bien que la face et le cou soient des régions exposées au soleil, le faible taux d’albinisme dans notre série amène à incriminer d’autres facteurs de risque tel l’augmentation des pratiques de dépigmentation dans la population noire. Il faut noter le fort contingent d’adénopathies cervicales malignes primitives dans notre série (18%) confirmant ainsi la tendance à l’augmentation des lymphomes ORL et CMF, ce qui fait que le spécialiste des VADS va être plus fréquemment confronté à cette pathologie [15].

## Conclusion

Les cancers ORL et CMF sont relativement fréquents au Togo. Les hommes sont les plus touchés mais il y a une forte proportion de femmes. L’alcoolotabagisme a été le facteur de risque majeur et les localisations les plus fréquentes ont été la cavité buccale, le pharynx et le tissu lymphoïde. Les cancers du massif facial, du cavum et de la peau faciale sont relativement rares au Togo. La nature histologique des cancers était largement dominée par le carcinome épidermoïde, suivi du LNH puis du carcinome papillaire thyroïdien.

### Etat des connaissances actuelles sur le sujet

- Sur le plan épidémiologique, la répartition par sexe dans le cas des cancers de la sphère ORL et surtout des voies aérodigestives supérieures est marquée par une disproportion entre hommes et femmes;
- La prédominance masculine est rapportée par la plupart des études;
- Sur le plan histologique, plusieurs données de la littérature s’accordent sur la rareté des cancers thyroïdiens et aussi sur la prédominance du carcinome mucoépidermoïde dans les cancers de la parotide.

### Contribution de notre étude à la connaissance

- Sur le plan épidémiologique, il y a eu une forte proportion de femmes présentant un cancer de la sphère ORL et surtout une prédominance féminine des cancers de la cavité buccale bien que la cavité buccale fasse partie des voies aérodigestives supérieures;
- Sur le plan histologique, l’adénocarcinome thyroïdien a été le 3^ème^ cancer en terme de fréquence dans notre étude et le carcinome épidermoïde a été fréquent dans les cancers de la parotide;
- Cette étude est une première du genre se déroulant dans le seul service de référence de prise en charge des cancers ORL et cervico-maxillo-faciaux au Togo, elle constitue ainsi une base de données pouvant servir pour des études ultérieures et pour la mise en place d’un registre des cancers.
